# Using benchmarked lung radiation dose constraints to predict pneumonitis risk: Developing a nomogram for patients with mediastinal lymphoma

**DOI:** 10.1016/j.adro.2018.03.005

**Published:** 2018-04-24

**Authors:** Chelsea C. Pinnix, Jinhai Huo, Sarah A. Milgrom, Zeinab Abou Yehia, Michelle Fanale, Yasuhiro Oki, Bouthaina S. Dabaja, Grace L. Smith

**Affiliations:** a Department of Radiation Oncology, The University of Texas MD Anderson Cancer Center, Houston, Texas; b Health Services Research, The University of Texas MD Anderson Cancer Center, Houston, Texas; c Lymphoma/Myeloma Medical Oncology, The University of Texas MD Anderson Cancer Center, Houston, Texas

## Abstract

**Purpose:**

We identified lung dosimetric constraints to assist in predicting the radiation pneumonitis (RP) risk in patients with mediastinal lymphoma and then identified the clinical prognostic factors that were associated with the achievement of key dosimetric constraints.

**Methods and Materials:**

In 190 patients who received mediastinal intensity modulated radiation therapy, we used univariate χ^2^ and multivariate logistic models to identify the predictors of RP and achievement of lung dose-volume histogram (DVH) constraints and build a predictive nomogram for RP.

**Results:**

An increased risk of RP was strongly associated with mean lung dose (MLD) > 13.5 Gy (odds ratio [OR]: 8.13; 95% confidence interval [CI], 3.01-21.93; *P* < .001) and the percent of lung volume receiving ≥5 Gy (V5) > 55% (OR: 7.01; 95% CI, 2.94-16.72; *P* < .001). Therefore, patients had low RP risk (8%) if both MLD ≤13.5 and V5 ≤55 constraints were achieved, moderate risk (24%) if only MLD was achieved, and the highest risk (48%) if MLD was not achieved. Deep-inspiration breath-hold (DIBH) technique during treatment strongly prognosticated achieving MLD and V5 DVH constraints (OR,3.88; 95% CI, 1.84-8.19; *P* < .001). Specifically, 86% of patients who were treated with DIBH versus 63% without DIBH achieved DVH constraints (*P* < .001). This translated into a “number needed to treat” with DIBH of 4 patients to enable 1 additional patient to achieve both constraints. In comparison, the clinical characteristics were marginal prognosticators: DVH constraints were more likely achieved in nonbulky disease (OR: 3.01; 95% CI, 0.89-4.53; *P* = .09) and patients who had not previously received salvage chemotherapy (OR, 2.44; 95% CI, 0.98-6.11; *P* = .06). Nomogram-predicted risks of RP ranged from 4% to 60% on the basis of MLD and V5, total radiation dose, and use of salvage chemotherapy.

**Conclusions:**

Achieving mean lung and V5 DVH constraints is critical to reduce RP risk in patients with lymphoma who receive mediastinal intensity modulated radiation therapy. The use of the DIBH technique is a promising risk-modifying treatment approach in patients with mediastinal lymphoma and especially in patients with a history of nonmodifiable risk factors for RP such as bulky disease and salvage chemotherapy.

**Summary**

We developed a nomogram to predict radiation pneumonitis (RP) risk in patients with mediastinal lymphoma after intensity modulated radiation therapy. RP risk was predicted on the basis of whether mean lung dose (≤13.5 Gy) and the percent of lung volume receiving ≥5 Gy (≤55%) criteria could be achieved. The deep-inspiration breath-hold technique during treatment strongly prognosticated achieving these dose-volume histogram criteria (odds ratio: 3.88; 95% confidence interval, 1.84-8.19; *P* < .001), which translated into a “number needed to treat” with deep-inspiration breath-hold of 4 patients to enable 1 additional patient to achieve both dose-volume histogram criteria. The nomogram-predicted RP risks ranged from 4% to 60%.

Alt-text: Unlabelled box

## Introduction

Avoiding irradiation of normal tissue is a fundamental component of high-quality radiation therapy, benchmarked through the use of dosimetric dose-volume histogram (DVH) constraints that were established to minimize toxicity.^1^, ^2^ For patients undergoing mediastinal radiation for lymphoma, the goal in using lung dosimetric constraints is to reduce the risk of radiation pneumonitis (RP), which is a relatively common and occasionally lethal side effect of thoracic irradiation. Patients with mediastinal lymphoma are at risk for RP with a risk of approximately 8% to 15% reported in prior studies.^3^, ^4^

Several nonmodifiable disease factors appear to influence RP risk. Patients with bulky disease or relapsed/refractory lymphoma seem to be especially vulnerable to RP, probably from the exposure to several lines of cytotoxic chemotherapy and targeted agents with known risks of lung damage.^5^, ^6^ Yet, these dosimetric benchmarks can be difficult to achieve in patients with mediastinal lymphoma due to the often large thoracic radiation target volumes and corresponding volumes of normal lung that are exposed to falloff radiation doses.

The magnitude of risk reduction of RP that is associated with achieving lung dosimetric constraints has not been well quantified. Also unknown is whether the risk of RP for patients with nonmodifiable adverse intrinsic disease characteristics can be reduced by meeting dosimetric constraints. We sought to address these knowledge gaps by analyzing a large group of patients who had received intensity modulated radiation therapy (IMRT) for mediastinal lymphoma. To develop an approach to predict RP risk in these patients, we sought to identify the lung dosimetric constraints that are associated with RP. Second, we sought to identify modifiable and nonmodifiable clinical prognostic factors that are associated with the achievement of these dosimetric constraints to reduce the risk of RP. These findings supported the development of a nomogram for RP risk after radiation therapy for lymphoma that is located in the mediastinum.

## Methods and materials

### Study population and clinical covariates

We retrospectively reviewed the records of 190 consecutive adult patients who received mediastinal IMRT for Hodgkin or non-Hodgkin lymphoma at our institution between 2009 and 2014. We abstracted patient characteristics including clinical covariates (ie, sociodemographic characteristics, comorbidity, histology, Ann Arbor disease stage, and bulky disease [defined as a conglomerate nodal mass of >10 cm on axial computed tomography imaging]) and treatment characteristics (ie, radiation dosimetric variables, use of a motion-management technique called deep-inspiration breath-hold [DIBH], and chemotherapy). Lung dosimetric information was obtained from the electronic radiation treatment plans.

### Radiation treatment techniques and planning

All patients underwent computed tomography-based simulation and IMRT treatment planning. The details of the simulation, DIBH, and IMRT are found in the Supplementary Methods section M1.^7^, ^8^, ^9^

### Radiation pneumonitis

Acute symptomatic RP was based on pulmonary symptoms up to 12 months from radiation treatment without evidence of other potential etiologies (eg, infectious pneumonia) and scored in accordance with the Radiation Therapy Oncology Group (RTOG) acute radiation morbidity scoring criteria.^10^ Grade 1 RP was defined as the development of mild dyspnea on exertion or dry cough, grade 2 RP as persistent cough that requires narcotic or antitussive agents as well as dyspnea on minimal exertion, and grade 3 as severe cough that is unresponsive to narcotic or antitussive agents, dyspnea at rest, or clinical or radiographic evidence of acute pneumonitis for which intermittent oxygen or steroidal medications may be required. Grade 4 RP was defined as severe respiratory insufficiency that requires continuous oxygen or assisted ventilation. Patients with radiographic evidence of radiation injury in the treatment field in the absence of pulmonary symptoms were not considered to have RP.

Medical records were reviewed and RP grades assigned by a board-certified radiation oncologist. All grades of RP were included in this analysis but we limited patients with grade 1 RP only to those who were clinically symptomatic. Time to RP was defined from the last day of IMRT to the date of development of pulmonary symptoms. No patients were lost to follow-up during this study period.

### Achievement of dosimetric constraints

Benchmarked dosimetric constraints to reduce the risk of RP (established to reduce symptomatic RP in published studies) were mean lung dose (MLD) ≤ 13.5 Gy, V20 (volume of lung that receives radiation dose ≥20 Gy) ≤ 30%, V15 ≤35%, V10 ≤40%, and V5 ≤55%.^5^, ^6^ These thresholds were derived on the basis of prior studies^5^, ^6^ as well as empiric testing of sensitivity, specificity, and receiver operating characteristics at 5% intervals for every volume threshold that is used in typical practice (V5, V10, V15, etc.).

Each dosimetric criterion was tested in univariate analyses as a dichotomous variable (achieved vs not achieved) for its association with RP. Based on the strength and magnitude of the univariate associations, subsequent nomogram analyses focused on V5 and MLD.

#### Lung dose-volume score derivation

To establish a variable that combines dosimetric constraints for V5 and MLD, we compared 2 possible V5- and MLD-based variables. We tested the 2-level variable of achieving versus not achieving 2 key constraints (MLD ≤13.5 Gy and V5 ≤55%). We also tested a 3-level variable on the basis of MLD and V5 constraints: Achieving both key dosimetric constraints, achieving MLD ≤13.5 Gy only, or not achieving MLD constraint (regardless of achieving the V5 constraint or not). On the basis of optimal model goodness-of-fit, the 3-level outcome was used for the nomogram development and translated into an ordinal lung dose volume (LDV) score. Whether or not the treatment plan achieved the V5 and MLD constraints was used to define the LDV score (defined as achievement of both constraints: good risk; MLD only: moderate risk; and not achieving the MLD constraint: poor risk).

### Statistical analysis

Our first analytic objective was to test the association of each dosimetric criterion with RP risk in individual logistic models that were adjusted for clinical covariates to select the most statistically significant dosimetric criteria for inclusion in the subsequent nomogram development. Our second objective was to identify the prognostic factors that were associated with the achievement of dosimetric constraints using the 2-level outcome (ie, achieving vs not achieving MLD ≤13.5 Gy and V5 ≤55%). Univariate Pearson χ^2^ and a parsimonious multivariate logistic model^11^ identified the significant risk factors of this outcome including testing the association with DIBH evaluated as an independent variable. The number needed to treat with DIBH to achieve all dosimetric constraints was calculated on the basis of the difference in absolute frequencies of achieving dosimetric constraints in patients who were treated with and without DIBH. The Hosmer and Lemeshow goodness of fit of each logistic model was assessed.

Our third objective was to derive a nomogram to predict RP risk. The included covariates were based on statistical (*P* < .25) or clinical significance,^5^ Hosmer and Lemeshow goodness of fit, and the Akaike information criterion for a parsimonious model selection with adequate fit.^11^ To improve the clinical utility of the nomogram in various practice settings, 2 separate models were derived for patients who were treated with and without DIBH given the heterogeneity in radiation-therapy practices and understanding that not all facilities may use DIBH. Each final model included a total of 3 covariates. There were no missing model covariates for all patients. The analyses were conducted with SAS statistical software (SAS Institute, Cary, NC) and assumed a 2-tailed alpha of .05.

Our fourth objective was to internally validate the model using bootstrapping to estimate models' bias- or overfitting-corrected predictive accuracy as evaluated with the concordance (C) index. The bootstrap-corrected odds ratios (OR) were based on 1000 bootstrap samples.

Calibration curves plotted the average estimate against the corresponding nomogram-predicted RP risk to evaluate the performance of the nomogram on the basis of the logistic model from which it was derived, which also confirmed that the selected parameters were chosen on the basis of an optimal model fit. The calibration curve and C index were built with the RMS software package in R.^12^ This analysis was exempted by the institutional review board.

## Results

### Patient characteristics

Of the 190 patients with lymphoma who received mediastinal IMRT, 136 patients (71%) had Hodgkin lymphoma, 156 patients (82%) stage I/II disease, and 115 patients (61%) bulky disease (>10 cm). Forty-four patients (23%) received salvage chemotherapy for relapsed/refractory lymphoma and all but one of these patients underwent stem cell transplantation. The median radiation dose was 30.6 Gy (interquartile range, 30.6-36.0). A majority of the patients (110 patients [58%]) were treated with DIBH. The median follow-up time was 23.8 months (Range, 4.8-69.8 months).

### Radiation pneumonitis risk

**Figure 1:**
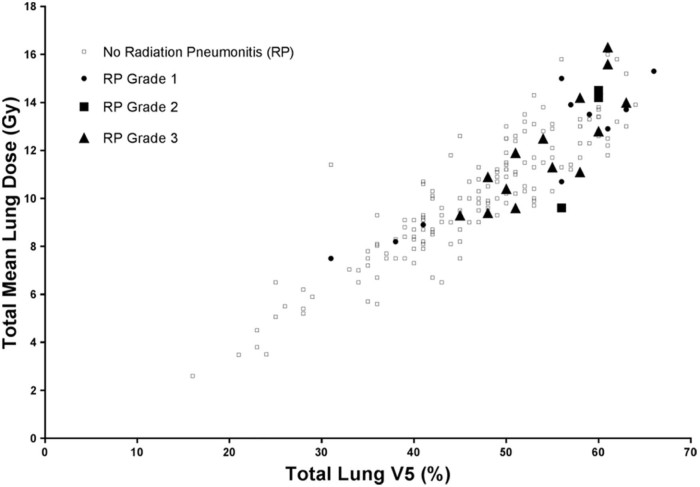
Mean lung dose, percent of lung volume receiving ≥5 Gy, and grades 1 to 3 radiation pneumonitis.

Symptomatic RP occurred in 27 patients (14.2%) and were graded in severity as 10 events of grade 1, 3 events of grade 2, and 14 events of grade 3 (Suppl. Table S1; available as supplementary material online only at https://doi.org/10.1016/j.adro.2018.03.005). With regard to the grade 3 events, 13 of 14 events were based on a requirement for steroidal medications and only 1 patient with a grade 3 event required oxygen. The strongest dosimetric prognostic factors that were associated with RP were MLD and V5 (ie, percent lung volume receiving ≥5 Gy; Fig 1). Specifically, compared with patients whose plans met the dosimetric constraints of MLD ≤13.5 Gy, patients who received an MLD >13.5 Gy demonstrated higher odds of RP (OR: 8.13; 95% confidence interval [CI], 3.01-21.93; *P* < .001).

RP incidence was 10% in patients with MLD ≤13.5 Gy versus 48% in patients with MLD >13.5 Gy. Compared with patients who met V5 ≤55%, those patients with V5 >55% also demonstrated higher odds of RP (OR: 7.01; 95% CI, 2.94-16.72; *P* < .001). RP risk was 8% in patients with V5 ≤55% versus 36% in patients with V5 >55%. The univariate associations of other dosimetric parameters with RP are reported in Supplementary Results section R3.

**Figure 2:**
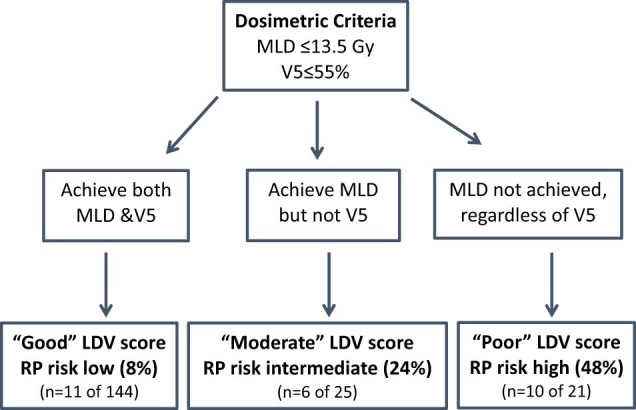
Derivation of lung dose volume (LDV) score on the basis of optimal goodness-of-fit for percent of lung volume receiving ≥5 Gy and mean lung dose dosimetric constraints to predict radiation pneumonitis.

RP incidence for combined bins of these dosimetric parameters were also identified. LDV constraints reflected the RP risks that were associated with potential combinations of MLD and V5 constraints (Fig 2): Good risk LDV score (ie, plans achieved MLD ≤13.5 Gy and V5 ≤55%) conferred an estimated RP risk of 8%, moderate LDV score (ie, achieving only MLD ≤13.5 Gy) had an estimated risk of 24%, and poor LDV score (ie, plans did not achieve MLD regardless of achieving the V5 constraint) had a risk of 48%.

### Prognostic factors that are associated with achieving dosimetric constraints

Seventy-six percent of patients achieved both dosimetric constraints that were associated with a reduced risk of RP (MLD ≤13.5 Gy and V5 ≤55%). On univariate analysis, patients were more likely to achieve both constraints with DIBH. Specifically, 86% of patients who were treated with DIBH (94 of 110 patients) achieved both constraints versus 63% of patients who were treated without DIBH (50 of 80 patients; *P* < .001). This translated into a “number needed to treat” with DIBH of 4 patients to enable 1 additional patient to achieve both constraints.

The association between use of DIBH and achieving dosimetric constraints persisted in the multivariate model that was adjusted for clinical characteristics (OR: 3.88; 95% CI, 1.84-8.19; *P* < .001). Use of DIBH remained the strongest predictor of achieving benchmarked constraints but baseline disease characteristics were of marginal influence. Constraints were marginally more likely to have been achieved if patients had nonbulky disease (OR: 3.01; 95% CI, 0.89-4.53; *P* = .09) or never required salvage chemotherapy (OR: 2.44; 95% CI, 0.98-6.11; *P* = .06; Table 1, Table 2).

**Table 1:** Baseline and treatment characteristics stratified by achievement of benchmarked dosimetric criteria (mean lung dose ≤13.5 Gy and percent of lung volume receiving less than 5 Gy ≤55%)

Characteristic	Total	Yes	%	No	%	*P*-value
Sex						
Female	108	78	54.2	30	65.2	.188
Male	82	66	45.8	16	34.8	
Ethnicity						
White	137	105	72.9	32	69.6	.958
African-American	14	10	6.9	4	8.7	
Hispanic	26	19	13.2	7	15.2	
Other	13	10	6.9	3	6.5	
Tumor histology						
Non-Hodgkin lymphoma	56	41	28.5	15	32.6	.592
Hodgkin lymphoma	134	103	71.5	31	67.4	
Disease stage						
I	19	17	11.8	2	4.4	.447
II	138	104	72.2	34	73.9	
III	14	10	6.9	4	8.7	
IV	19	13	9.0	6	13.0	
Asthma/COPD						
No	175	131	91.0	44	95.7	.306
Yes	15	13	9.0	2	4.4	
ABVD						
No	68	49	34.0	19	41.3	.370
Yes	122	95	66.0	27	58.7	
Smoking history						
No	151	114	79.2	37	80.4	.853
Yes	39	30	20.8	9	19.6	
Bulky disease (>10 cm)						
No	75	63	43.8	12	26.1	.033
Yes	115	81	56.3	34	73.9	
Required salvage chemotherapy						
No	146	119	82.6	27	58.7	.001
Yes	44	25	17.4	19	41.3	
Bleomycin toxicity						
No	156	118	81.9	38	82.6	.919
Yes	34	26	18.1	8	17.4	
Deep-inspiration breath-hold						
No	80	50	34.7	30	65.2	< .001
Yes	110	94	65.3	16	34.8	
History of stem cell transplant						
Yes	43	24	16.7	19	41.3	.001
No	147	120	83.3	27	58.7	
Brentuximab						
No	170	133	92.4	37	80.4	.022
Yes	20	11	7.6	9	19.6	

ABVD, adriamycin, bleomycin, vinblastine, and dacarbazine; COPD, chronic obstructive pulmonary disease.

**Table 2:** Multivariate predictors of achieving benchmarked mean lung dose and percent of lung volume receiving <5 Gy dosimetric criteria

Effect	OR	95% CI		*P*-value
Deep-inspiration breath-hold				
No	1.00			
Yes	3.88	1.84	8.19	< .001
Bulky disease (>10 cm)				
No	3.01	0.89	4.53	.09
Yes	1.00			
Required salvage chemotherapy				
No	2.44	0.98	6.11	.06
Yes	1.00			
Radiation treatment dose (per Gy)	0.95	0.87	1.03	.23

CI, confidence interval; OR, odds ratio.

### Nomogram to predict radiation pneumonitis risk

The nomogram predicted a range of RP risks on the basis of LDV score, prescribed radiation dose, and history of salvage chemotherapy for relapsed/refractory disease. RP risks ranged from as low as 4% for patients with the most favorable characteristics to 60% for patients with the least favorable characteristics (Fig 2). For example, if a patient had an LDV score of good, received 20 Gy to the mediastinum, and had no history of salvage therapy for relapsed disease, the predicted RP risk was 4%. On the other hand, a patient with a poor LDV score who received >45 Gy and required salvage therapy had an estimated RP risk of up to 60%. However, for patients at the highest risk at baseline (otherwise expected to have an estimated RP risk of 60%), the estimated risk of RP could be reduced to 16% if a patient with similar adverse baseline characteristics could achieve a good LDV score.

**Figure 3:**
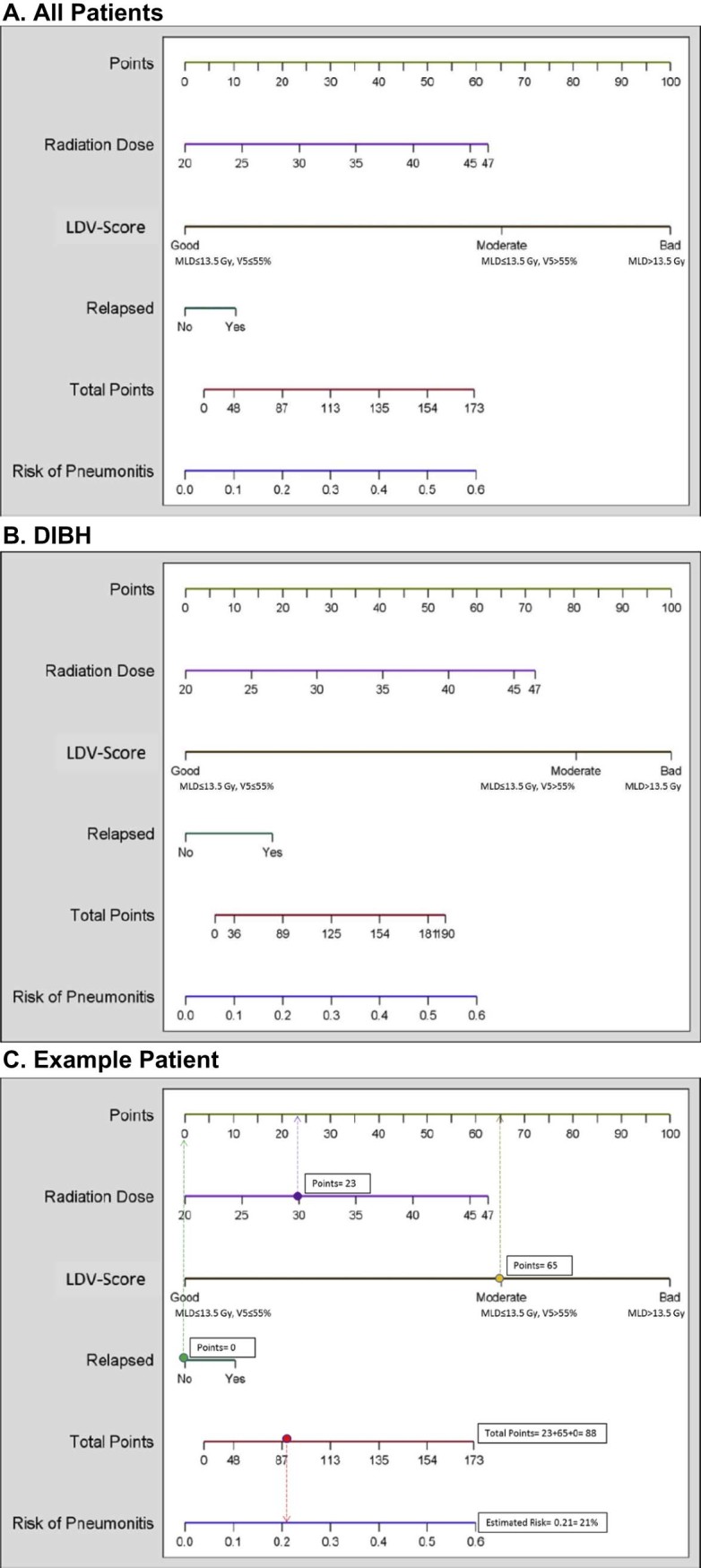
Nomogram to predict risk of radiation pneumonitis for patients with mediastinal lymphoma who were treated with intensity modulated radiation therapy. (A) All patients. Fit characteristics: For the above multivariate model, Hosmer-Lemeshow test *P* = .18 and Akaike Information Criteria 142.59 compared with a model including only lung dose volume score as a covariate, Hosmer-Lemeshow *P* = 1.0, and Akaike Information Criteria 140.34. (B) Patients treated with deep-inspiration breath-hold technique. Fit characteristics: For the above multivariate model, Hosmer-Lemeshow test *P* = .69 and Akaike Information Criteria 93.19 compared with a model including only lung dose volume score as a covariate, Hosmer-Lemeshow *P* = 1.0, and Akaike Information Criteria 90.45. (C) Example patient: A patient was treated without deep-inspiration breath-hold to 30 Gy. The plan achieved mean lung dose constraint but not percent of lung volume receiving ≥5 Gy constraint. The patient did not have relapsed mediastinal lymphoma. To calculate total points: 88, calculate the sum of points that are associated with the 1) patient's dose of 30 Gy: 23 points; 2) Lung dose volume score of moderate: 65 points; and 3) No relapse status: 0 points. Draw a straight line from total points calculated to find the risk of pneumonitis (eg, total points of 188 correlates with risk of 0.21 or 21%).

A separate nomogram model that was created for patients who had been treated with DIBH also demonstrated a similar range of RP risks and peaked at approximately 60% (Fig 3). Therefore, even in this subset of patients, lower RP risks could be achieved only if benchmarked dosimetric constraints were reached.

### Internal model validation

The model demonstrated accuracy to predict RP with a bootstrap-corrected C index of 0.73 (Suppl. Results Section R4; calibration curves with acceptable fit in Suppl. Fig. S1; available as supplementary material online only at https://doi.org/10.1016/j.adro.2018.03.005).

## Discussion

In patients who received contemporary mediastinal IMRT for lymphoma between 2009 and 2014, exceeding 2 benchmarked dosimetric constraints predicted an increased risk of subsequent pneumonitis: MLD >13.5 Gy and V5 >55%. The frequency of pneumonitis was approximately 14% in this cohort. Use of the DIBH technique during treatment significantly predicted achievement of these benchmarked constraints. The results suggest that DIBH is a promising tool that can affect the successful achievement of key dosimetric constraints for effective radiation treatment of lymphoma.

We also found a low “number needed to treat” with DIBH as only 4 patients needed to be treated with this technique to achieve the key dosimetric constraints per 1 additional patient, which underscores the potential clinical value of this treatment approach. This “number needed to treat” helps provide a data-driven framework for clinicians to understand and evaluate the potential risks, benefits, and investment of applying the practice of DIBH in IMRT for this patient group.

### Comparison with prior studies

In our study of patients who received IMRT to the mediastinum for lymphoma,14% experienced RP. This is similar to the incidence and distribution of grade 1 to 3 RP in a study of 92 patients who were treated at the Dana Farber Cancer Institute (DFCI).^6^ In that report, patients with Hodgkin lymphoma were treated with 3-dimensional conformal radiation therapy and 14% of patients experienced RP. In the DFCI study and the current report, grade 3 RP was relatively common and represented 38% and 52% of RP events, respectively. However, in contrast, in a study that was conducted at the Federico II University in Italy, 69 patients were treated with 3-dimensional conformal radiation therapy for Hodgkin lymphoma and 13% of patients developed RP.^3^ However, only 1 of 8 events (13%) was grade 3. In a report from St. Jude Children's Research Hospital of 99 pediatric patients with Hodgkin lymphoma, the incidence of RP was 10% with no grade 3 events.^4^

The higher relative frequency of grade 3 events in our study likely reflects variations in study definitions of toxicity grades. First, our study and the DFCI study both excluded grade 1 asymptomatic patients with radiographic changes alone but the St. Jude and Italian studies included such asymptomatic patients (and therefore increased the relative representation of grade 1 RP and concurrently decreased the relative representation of grade 3 RP in their samples). The purpose of excluding asymptomatic RP patients in our study was to improve the use of our analytic model and nomogram as a clinically relevant tool.

Second, grade 3 RP represented more severe toxicity in our study than in the St. Jude and Italian studies because grade 3 RP in those studies had a requirement for oxygen or severe symptomatic fibrosis with dense radiographic changes, respectively, which further decreased the relative representation of grade 3 RP in those samples. In contrast, only 1 grade 3 patient required oxygen in our study.

Another consideration is that our study and the DCFI study allowed for the inclusion of patients with refractory disease who have higher risks of RP that are associated with increased RT doses and exposure to chemotherapy and autologous stem cell transplantation. In contrast, the St. Jude and Italian studies included only patients with newly diagnosed disease in whom the risks of RP are known to be lower.^5^

The comparative importance of various dosimetric constraints continues to be debated and particularly in the current era of IMRT use. In prior studies of patients with lung cancer, MLD and V20 were established as predictive of RP among patients who were treated with 3-dimensional conformal radiation therapy.^13^, ^14^ However, as the use of IMRT for lung cancer and other disease sites increased, the potential added value of a V5 constraint to reduce RP was recognized.^15^, ^16^ In a single-institution review of IMRT for patients with early stage mediastinal Hodgkin lymphoma, the mean V5 was 75% and median MLD was 13.8 Gy but no patient developed grade ≥2 RP.^17^ A potential explanation for the low RP incidence may reflect the favorable risk characteristics of the study population: All patients were treated for limited-stage disease after a favorable response to the initial systemic therapy (more likely to fall into the best risk category in our nomogram).

In patients with locally advanced lung cancer, the recent findings from a secondary analysis of RTOG 0617 did not identify V5 as a significant predictor of RP.^18^ However, this analysis included both 3-dimensional conformal and IMRT treatment and did not stratify RP risks by technique. Conversely in a single-institution series from Denmark, the introduction of IMRT for patients with locally advanced lung cancer led to an increased incidence of severe RP. The use of a V5 constraint during the most recent phase of IMRT implementation in this group subsequently reduced the incidence.^16^

Dosimetric influences of radiation-induced lung injury are possibly distinct for patients with lung cancer and those with lymphoma who are treated with IMRT to the thorax. In our study of patients with lymphoma, the volume of lung that was exposed to low doses significantly affected RP risk. Attention to the V5 in treatment-plan evaluations has implications beyond acute toxicity for these patients. The reduction of the integral dose may also be desirable for patients with lymphoma because of the risk of developing secondary malignancy and thus potential long-term morbidity.^19^

### Implications

Our findings have practical clinical implications. Predicting RP risk for patients who receive mediastinal radiation for lymphoma is integrated in our nomogram that incorporates clinical and treatment factors. Our analysis suggests that nonmodifiable disease characteristics that initially have a strong influence on baseline elevated risk for RP (with adverse features that produce higher baseline risk) may be overcome by using DIBH and successful achievement of benchmarked dosimetric constraints. In fact, after adjusting for successful achievement of dosimetric constraints (ie, MLD ≤13.5 Gy and V5 ≤55%), baseline intrinsic disease factors became only marginally predictive of RP risk.

Therefore, our analysis also suggests that the discerning and judicious application of DIBH by radiation oncologists could help optimize the attainment of dosimetric constraints. DIBH is an attractive technique to improve dosimetry and permits lung expansion as well as respiratory motion control, which both contribute to the reduction of the amount of normal lung parenchyma that is exposed to radiation.^20^ Our findings confirmed that the DIBH technique was associated with more frequent achievements of V5 and MLD constraints in this patient group. However, DIBH in our study was not an independent protective factor for the outcome of RP development and the association was likely confounded by a variety of temporal trends in practice at our institution including an increased use of DIBH but also the concomitant application of higher doses for more refractory patients.

Predictive clinical tools can be useful to help physicians and patients make personalized risk assessments and guide important treatment decisions. For patients with lung cancer who were treated with 3-dimensional conformal radiation therapy, a nomogram that is based on combined data sets from RTOG 9311 and single-institution data^2^ showed that RP risk was most strongly influenced by MLD and radiation dose to the inferior lung. The value of refining such nomograms by disease site is to better personalize the prospective targeting of modifiable factors such as dosimetric parameters during radiation treatment.

### Limitations and future directions

A main limitation of our study is the need for external validation of our predictive model in additional patient cohorts and until external validation has been achieved, our model should be interpreted with caution. This is a challenging limitation because IMRT is not yet routinely used to treat lymphoma in some practice settings. However, we expect that IMRT will become more commonly used for such patients and we propose this as an area for future study.

Another consideration is our use of a uniform IMRT beam arrangement. In the current series, all patients were treated with strongly anterior/posterior and posterior/anterior-weighted IMRT beam planning^8^ (ie, butterfly technique) and the risk of RP for patients who are treated with IMRT without these restricted beam angles may be different. Conversely, the lung dose distribution is still distinct from standard anterior/posterior and posterior/anterior fields with particularly low-dose distributions that are higher than those found with a strict 3-dimensional planning approach. The risks of RP with other beam angle approaches or volumetric arc therapy may need additional investigation.

These limitations highlight the challenges in evaluation and validation of clinical outcomes for newer radiation technologies and techniques, which can quickly penetrate practice while outcomes data are generated. Our findings serve as initial clinical outcomes data for this treatment of mediastinal lymphoma in anticipation of iterative future validation of our predictive nomogram tool as data and follow-up from other sources emerge.

## Conclusions

We found that both clinical and dosimetric factors influenced the risk of RP in our cohort of patients with lymphoma. Adverse clinical factors may be mitigated by improvements in dosimetric parameters and the judicious use of DIBH during treatment. Our nomogram can be useful to initially guide clinicians using IMRT treatment in this patient group and prompt additional iterative external validation of this approach to RP risk stratification.
