# Supplementary material for: Using benchmarked lung radiation dose constraints to predict pneumonitis risk: Developing a nomogram for patients with mediastinal lymphoma
Source: Adv Radiat Oncol. 2018 Apr 24;3(3):372–81. doi: 10.1016/j.adro.2018.03.005 (PMC6128097; doi:10.1016/j.adro.2018.03.005)
Supplement: Appendix S1 — Supplementary Material. [file mmc1.pdf]

M1. ***Radiation Treatment Techniques and Planning (Detailed)***

All patients underwent CT-based treatment simulation while supine and immobilized in customized cradles. Patients were treated on a 10°–15° incline board to promote inferior-posterior displacement of the heart and mammary tissue (in female patients) as well as to facilitate DIBH.[5] Respiration was monitored with a video-based, noninvasive system that includes an infrared tracking camera and reflective marker (RPM [Real-time Position Management] System, Varian Medical Systems, Palo Alto, CA). Patients were coached on how to perform the DIBH and received feedback via video goggles to improve the stability and reproducibility of breath amplitude. At treatment simulation, 3-4 breath-hold gated non-contrast CT scans were acquired with axial 2.5-mm slices. The DIBH technique was used during treatment if the patient could tolerate and reproduce breath-holds during treatment simulation. When DIBH was not used, four-dimensional CT images were acquired to assess respiration-induced motion of the target volume, and the radiation target was delineated based on the motion of that target volume. Imaging data were transmitted to a Pinnacle treatment planning system (Phillips Healthcare) to allow contouring of the tumor target and surrounding organs at risk as well as treatment planning, as described below.

IMRT plans were generated with the Pinnacle treatment planning system. Co-planar 6-MV photons were used in a 5- to 7-beam anterior-posterior weighted "butterfly" technique.[6] Involved-site radiation therapy was used for target delineation.[7] IMRT was delivered by a linear accelerator with step-and-shoot multi-leaf collimation. Patients who could use DIBH during treatment completed sequential breath-holds, with one or two

breath-holds typically required for each beam angle. Low-dose CT-on-rails was used for daily image guidance (Varian Medical Systems).

The entire lung was also delineated on the treatment planning scans and included all pulmonary tissue regardless of the parenchymal lung tissue that was within the planning target volume. Dose-volume variables were recorded for each patient based on the total lung dose-volume histogram (generated by the Pinnacle system).

M2. *Radiation Pneumonitis (Detailed Definition)*

Grade 1 RP was defined as the development of mild dyspnea on exertion or dry cough; grade 2 RP as persistent cough requiring narcotic or antitussive agents and dyspnea on minimal exertion; and grade 3 severe cough unresponsive to narcotic or antitussive agents; dyspnea at rest; or clinical or radiographic evidence of acute pneumonitis for which intermittent oxygen or steroids may be required. Grade 4 RP was defined as severe respiratory insufficiency requiring continuous oxygen or assisted ventilation. Patients with radiographic evidence of radiation injury in the treatment field in the absence of pulmonary symptoms were not considered to have RP. Medical records were reviewed and RP grades assigned by a board-certified radiation oncologist.

R3. *Radiation Pneumonitis Risk (Univariate Associations)*

Univariate associations of other dosimetric parameters with RP, though significant, did not demonstrate as high a magnitude of risk: For V10 >40% OR=4.47, 95% CI 1.92-10.42, $P<0.001$; V15 >35% OR=4.89, 95% CI 2.06-11.60, $P<0.001$; and V20 >30% OR=4.29, 95% CI 1.67-11.05, $P=0.003$.

R4. *Internal Model Validation*

Using patients with “good” LDV score as the referent category for predicting the outcome of RP, patients with a “moderate” LDV score had a pooled OR of 4.15 (95% CI 1.28–11.28) and those with a “poor” LDV score had a pooled OR of 8.95 (95% CI 3.00–26.76).
