# Supplementary material for: Using benchmarked lung radiation dose constraints to predict pneumonitis risk: Developing a nomogram for patients with mediastinal lymphoma
Source: Adv Radiat Oncol. 2018 Apr 24;3(3):372–81. doi: 10.1016/j.adro.2018.03.005 (PMC6128097; doi:10.1016/j.adro.2018.03.005)

Supplemental Table S1. Radiation Pneumonitis According to Purpose of Therapy

	All Patients (n=190)		No. Receiving Consolidative RT (n=146)		No. with Relapsed or Refractory Disease (n=44)		
**Pneumonitis**	N	%	N	%	N	%	*P* Value
**None**	163	85.8	130	89.0	33	75.0	0.03
**Any**	27	14.2	16	11.0	11	25.0	
**Grade 1**	10	5.3	7	4.8	3	6.8	0.70
**Grade 2**	3	1.6	1	0.7	2	4.6	0.13
**Grade 3**	14	7.4	8	5.5	6	13.6	0.10

Supplemental Fig. S1. Nomogram model calibration curves. The ideal fit, where nomogram-predicted probability of RP (x-axis) is equal to the observed probability of RP, is represented by the dashed line.

A.

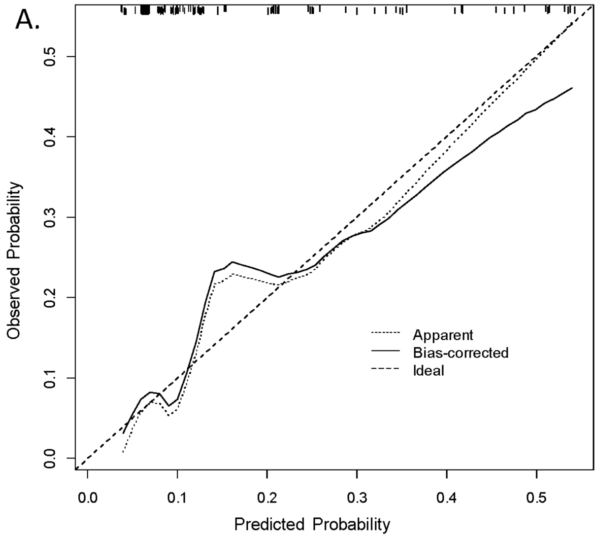

Supplement: Appendix S2 — Supplementary Table and Figure. [file mmc2.pdf]
